# Role of Bone Turnover Markers and Bone Mineral Density in Monitoring Bone Health at One- and Three-Year Post-antiresorptive Therapy Initiation in Patients With Osteoporosis: A Retrospective Cohort Study

**DOI:** 10.7759/cureus.105675

**Published:** 2026-03-22

**Authors:** Ahmed T Al-Jumaily, Batseba Bereket Araya, Ashiyana Malil, Chakraborty Oung, Flavian Joseph, Tripti Joshi

**Affiliations:** 1 Hospital Medicine, Central Coast Local Health District, Gosford, AUS; 2 Hospital Medicine, Gold Coast Hospital and Health Service, Gold Coast, AUS; 3 Endocrinology, Diabetes, and Metabolism, Central Coast Local Health District, Gosford, AUS

**Keywords:** antiresorptive, bone mineral density, bone turnover markers, ctx, denosumab, oral bisphosphonates, osteoporosis, p1np, teriparatide, zoledronic acid

## Abstract

Background

The gold-standard assessment of bone health is bone mineral density (BMD) measurement using dual-energy X-ray absorptiometry (DEXA); however, this modality is limited in its ability to detect short-term changes. Bone turnover markers (BTMs), specifically serum C-terminal telopeptide of type I collagen (CTX) and procollagen type I N-terminal propeptide (P1NP), offer the advantage of more frequent monitoring, yet their reliability as surrogate markers of bone health remains uncertain. This study investigates the relationship between BTMs and BMD during osteoporosis treatment.

Methods

Records of 214 patients treated at the Central Coast Osteoporosis Refracture Prevention (ORP) Clinic between June 2018 and May 2023 were reviewed. The relationship between BMD change (∆BMD) and changes in the levels and ratio of BTMs (∆BTM), specifically the propeptide of type I procollagen (P1NP) and the b-C-terminal telopeptide of type I collagen (CTX), at one-year and three-year post-treatment initiation was assessed. Simple statistical analysis and evaluation of the correlation coefficient were performed, including group stratification into clinically stable and clinically positive ∆BMD. A secondary analysis also stratified patients into those treated with IV versus oral bisphosphonates, using analysis of variance to compare the average change in BTM.

Results

Correlations between ∆BMD at all sites and ∆BTMs or the P1NP:CTX ratio were weak and not significant at one year. BTMs generally decreased with BMD improvement. There was no significant difference in BTM change over one to three years between patients with BMD improvement and those with BMD stability. However, ∆CTX was significantly greater in the improvement group in zoledronic acid (ZA)-medicated patients. These patients also had significant BMD changes at the hip and spine. ∆BTMs reached the clinical least significant change (LSC) with both medications.

Conclusions

Changes in BTMs and the P1NP:CTX ratio appear poorly correlated with medium- to long-term BMD changes. BTMs could potentially indicate therapeutic efficacy early in treatment, CTX perhaps more reliably than P1NP. Attainment of the LSC may more accurately predict BMD improvement than the magnitude of ∆BTMs. ZA may improve BMD more than oral bisphosphonates.

## Introduction

Osteoporosis is an imbalance in the process of bone resorption and formation that leads to low bone mineral density (BMD), microarchitectural disruptions, and skeletal fragility [1]. This imbalance in bone remodelling processes can be measured using bone turnover markers (BTMs) [1]. There has been greater clinical interest in the assessment of treatment efficacy regarding the use of BTMs, BMDs, and correlations between them. Multiple studies, including the prominent HORIZON-Pivotal Fracture Trial, which assessed osteoporotic patients treated with bisphosphonates, have proposed that the lower levels of BTMs found in treated patients may indicate a decreased fracture risk [2]. However, the relationships between BTMs, BMD, and the various anti-osteoporotic therapies need to be further explored to better guide monitoring of bone health and therapeutic efficacy.

In Australia, osteoporosis is estimated to affect 23% of women and 6% of men over 50 - figures that continue to increase [3]. Due to the decrease in bone density, patients are at high risk of fractures, which can cause significant pain and decrease functional status [2]. A 2012 study found that patients with osteoporosis in Australia and other countries had increased difficulty with self-care, activities, and persistent pain. Resulting in loss of independence, changes in family dynamics, and decreased social interaction, which lead to declining emotional health [4]. Additionally, there is a staggering financial impact on the healthcare system; the associated costs of fracture-associated hospitalisation, rehabilitation, imaging, and other aspects of the disease burden totalled approximately $3.44 billion in 2017 [5].

The two groups of BTMs are markers of bone formation (chiefly procollagen type I N-terminal propeptide (P1NP)) and markers of bone resorption (chiefly C-terminal cross-linking telopeptide of type I collagen (CTX)) [1]. There is a large intrapersonal, pre-analytical, and analytical variability regarding their use. However, there is growing evidence base for BTMs’ association with fracture presence and healing in relation to osteoporosis management in recent years [6].

BTMs increase with ageing and pathological states (e.g., fracture healing) and with metabolic bone disease, indicating increased bone turnover and deterioration of bone microarchitecture [7]. Veitch et al. showed that following tibial shaft fractures, CTX levels rose dramatically within the first two weeks and remained elevated for 24-30 weeks [7]. P1NP levels were maximal at 12 weeks post-fracture and then remained elevated for a further 12 weeks [8]. In orthogeriatric patients who were hospitalised with both vertebral and nonvertebral fractures, the P1NP:CTX ratio was inversely proportional to the rate of nonvertebral fracture and independently indicative of fracture presence [7,9], consistently being decreased post-fracture. In contrast, an observational study found that in premenopausal women with distal radius fractures, P1NP levels increased by more than CTX [10]. Thus, in patients without osteoporosis, the P1NP:CTX ratio appears to be increased post-fracture, but the significance of this decreased P1NP:CTX ratio in osteoporosis remains to be explored.

The association between BTMs and BMD is controversial, due primarily to large discrepancies in findings and imprecise assays. Currently, the use of BMD to predict minimal trauma fractures is the consensus standard, and the role of BTMs in this context has not been established. Chiefly, P1NP as a reflection of bone formation and CTX as a reflection of bone resorption can reflect changes in bone health and changing trends in BMD. Furthermore, they can be used to monitor the efficacy of drug therapies with adequate suppression - at least 20% and 30%, respectively, with variability due to drug and dose - indicating low bone turnover [11].

Modifiable and non-modifiable risk factors of osteoporotic fracture have been extensively studied and include factors such as age, body mass index (BMI), menopausal status, and smoking status [12]. However, their comparative effects at baseline in response to antiresorptive therapy and their correlation with turnover markers are less thoroughly understood. A cross-sectional study comparing BTMs of young adult smokers to non-smokers showed a decrease in CTX values for smokers [13]. Similar results were found regarding smoking’s suppression of CTX in a study by Al-Bashaireh et al. [14], which also found a correlation between smoking and decreased BMI, linking decreased BMI to BTM suppression [14].

BMD is usually measured at the lumbar spine, proximal femur, and femoral neck using a dual-energy X-ray absorptiometry (DEXA) scan [15]. This cannot independently identify patients at risk of fracture, as other risk factors (e.g., smoking status, inflammatory disorders, and the use of certain medications and BTMs) are involved [16]. An extensive body of research investigates the correlation between BMD and BTM. In response to antiresorptive agents, CTX levels have been shown to decrease rapidly and significantly, whereas P1NP levels decrease more slowly and to a lesser degree. Both decreases are associated with increased BMD levels [17]. A 2020 systematic review of 22 articles found a strong negative correlation of BTM changes with BMD post-antiresorptive therapy [16]. Randomised controlled trials confirm this, showing that in postmenopausal women with osteoporosis, bisphosphonates and denosumab suppress BTMs and increase BMD [16-20].

Bisphosphonates (administered orally or intravenously) are the first-line treatment in osteoporosis, inhibiting osteoclastic bone resorption by attaching to hydroxyapatite binding surfaces on bone. There is strong evidence regarding bisphosphonate suppression of BTMs. A 2021 meta-analysis reviewed 16 studies in which osteoporosis patients were treated with bisphosphonates post-fracture. Oral bisphosphonates significantly decreased BTMs and increased BMD without delaying fracture healing [21]. By inhibiting reabsorption, they caused greater suppression of resorption than of formation markers, indicating an increased P1NP:CTX ratio [21]. However, this ratio was not directly measured in any individual study, nor was it looked at in any specific time frame. A 2009 RCT of 1053 post-menopausal women treated with alendronate and risedronate found that reduction in BTMs began as early as three months after initiation of therapy and was maintained at 6 and 12 months [22]. It also showed that the rate of BTM suppression decreased significantly over time. However, in a more recent but smaller RCT following 53 Chinese women with osteoporosis, the rate of suppression with alendronate was either stable (CTX) or increasing (P1NP) [23]. It remains unclear whether BTM thresholds result in a plateau in fracture risk improvement following bisphosphonate therapy [22-24].

The international HORIZON Refracture Trial studied the effect of zoledronic acid (ZA), an intravenously administered bisphosphonate, on 7765 postmenopausal women. Overall, ZA was effective in suppressing BTMs, although the number of patients included in the P1NP assessment was not reported [25]. This finding has since been supported by various trials [24,26-28]. Studies consistently attribute greater BTM suppression to ZA than to oral bisphosphonates [24,27,28]. Although oral bisphosphonates may have reduced gastrointestinal absorption when taken with food, the route does not affect their bioavailability. This greater effect of ZA on BTMs may therefore be explained by its higher affinity for hydroxyapatite than that of the oral bisphosphonates and by better patient compliance due to its annual intravenous administration [24,29]. Most studies indicate an increased P1NP:CTX ratio in ZA [26,28], and two studies assessing the use of ZA in osteoporotic patients found direct links between decreased BTMs and a reduced risk of refracture. In determining the efficacy of these drugs, clinicians take measurements at the hip, vertebrae, and wrist over time. However, their data are minimal regarding which bisphosphonate is best for each area. A meta-analysis of 36 studies, particularly focusing on fracture prevention, found that ZA was preferred for all fracture prevention, and alendronate was additionally preferred for hip fractures [8].

There are conflicting findings regarding the correlation between BTMs and BMD during treatment. Few studies have directly compared turnover markers in the context of ZA with oral bisphosphonates, and as a head-to-head comparison is the best method of assessing relative efficacy [22], this absence of evidence makes treatment decisions somewhat ambiguous. Furthermore, there is conflicting evidence for each drug’s effect on BTMs and on the P1NP:CTX ratio. This ratio may be useful as another indicator of bone health, considering both bone resorption and formation. There is also limited data regarding the optimal duration of therapy and the ability of markers to reflect this.

We therefore aimed to investigate the correlation of BTMs with BMD in their use, indicating bone health and therapeutic responses in the context of oral and IV bisphosphonate treatment. This may guide future use of BTMs in monitoring osteoporosis and refracture risk.

Aims

The primary objective of this study was to retrospectively examine the correlation between changes in BTMs - specifically serum CTX and P1NP - and changes in BMD, as measured by DEXA, at one and three years following the commencement of anti-osteoporotic therapy in patients attending the Osteoporosis Refracture Prevention (ORP) Clinic, Endocrine Department, Central Coast Local Health District.

The secondary objectives were as follows: (1) to assess the degree of suppression of CTX and P1NP at one and three years following the commencement of anti-osteoporotic therapy; (2) to evaluate the utility of the P1NP:CTX ratio as a composite indicator of the relative balance between bone formation and resorption and its value in assessing therapeutic response; (3) to compare the short-term effects of oral bisphosphonates and ZA on BTMs and BMD; and (4) to identify any discernible trends in the time course of therapeutic effect between these two treatment modalities.

## Materials and methods

Study design

This was a five-year retrospective clinical audit examining the relationship between BTMs and BMD during anti-osteoporotic therapy. Data were obtained from the Central Coast ORP clinic and accessed through Electronic Medical Records (eMR). The study period extended from 1st June 2018 to 1st May 2023.

Ethics statement

The study protocol obtained ethics approval from the Northern Sydney Local Health District Human Research Ethics Committee and was approved for conduct at both Gosford and Wyong Hospitals (Reference: 2022/ETH01632). External ethics approval was registered with the University of Newcastle and the University of New England. As this was a retrospective audit of de-identified data, individual patient consent was not required.

Study population

Patients were identified following screening from hospital presentations with osteoporosis-related fractures and through referral from their respective general practitioner (GP) practices. Inclusion criteria were adult males and females aged 30 years or over, with either a radiological (T-score < −2.5) or clinical (history of at least one minimal trauma fracture (MTF)) diagnosis of osteoporosis, who were being treated with anti-osteoporotic monotherapy comprising oral bisphosphonates, ZA, teriparatide, or denosumab.

Patients were excluded if they did not have at least one set of follow-up results within the study period, had no confirmed diagnosis of osteoporosis, were less than 30 years old, were receiving an anti-osteoporotic agent outside of the four treatments specified, declined treatment, or had osteoporosis secondary to a specified set of medical conditions. These conditions included traumatic fracture, rickets or osteomalacia, renal osteodystrophy, Paget's disease of the bone, developmental skeletal disorders such as osteogenesis imperfecta, or skeletal malignancy. All patients who received anti-osteoporotic treatment at any point during the study period were included in the sample to minimise a loss-to-follow-up bias. For patients whose medical records were incomplete due to early termination of treatment or failure to re-present for follow-up, the reason for the lack of clinical information was recorded.

Sample size

Data were collected from 214 patients. Due to the small number of patients treated with denosumab (n = 18) and teriparatide (n = 1), these subgroups were not included in the medication subgroup analysis. Consistently, over 120 patients had results measured at both baseline and one year for all BTM and BMD measures.

Data collection

Data were collected through the PowerChart and Audit4 database eMR systems. A separate database using Microsoft Excel (Microsoft Corporation, Redmond, WA, USA) was created for data entry and storage. The following data were collected: (1) demographics and clinical characteristics: age, sex, ethnicity, BMI, smoking status, menopausal status, physical activity levels, and family history of osteoporosis (defined as an osteoporosis diagnosis in a parent); (2) treatment details: type of anti-osteoporotic therapy, onset of sentinel fracture, and number of fractures sustained during the study period; (3) bone turnover markers: baseline and follow-up serum levels of CTX and P1NP, measured using a consistent assay. BTM levels were assessed alongside BMD at baseline, and any measurements taken between the DEXA scans performed at the one- and three-year time points were also collected; (4) bone mineral density: BMD levels at baseline and follow-up by DEXA scan, the type of scanner used, and whether the same scanner was used at follow-up review; and (5) additional variables: relevant medical conditions outside of exclusion criteria, current or previous use of osteoporosis-inducing medications, vitamin D supplementation and levels at baseline and follow-up, and calcium supplementation.

Data on the number of refractures during the study period were also collected; however, this did not form a separate subgroup for assessment, as patient numbers were low (n = 11).

Prior anti-resorptive therapy use was recorded for all patients when available in the medical record. However, due to the small number of patients with prior anti-resorptive exposure in this cohort, reflecting the predominantly treatment-naïve referral population of the Central Coast ORP clinic, formal subgroup correlation analysis by prior therapy was not performed.

Study measures

BMD

All BMD values were obtained at the patient's local pathology centre or at Gosford Hospital, using either the Hologic Horizon DXA Platform (Hologic Inc., Marlborough, MA, USA) or the GE Lunar Prodigy (GE Healthcare, Madison, WI, USA). Where possible, measurements were recorded at L1-L4 for spine BMD, total hip for hip BMD, and at the 33% (one-third) radius for wrist BMD. L1-L4 and the 33% radius are the current clinically recommended measurement sites at the spine and wrist, respectively [30], and the total hip provides a superior indication of BMD changes over time [31]. However, some laboratories reported BMD only at specific alternate sites, including L2-L3 or another vertebral combination for the spine, particularly if an MTF at the relevant level meant that measurement at L1-L4 would yield misleading results. Similarly, some hip BMD measurements were only reported at the femoral neck. Where patients had BMD measured at both the left and right sides of the relevant site, the average of the two values was used. It should be noted that BMD values obtained from the Hologic and GE Lunar systems are not directly interchangeable without cross-calibration, and the use of different scanners between baseline and follow-up may introduce measurement variability. A clinically positive BMD change was defined as greater than a 3% change from the previous BMD, while a clinically stable BMD was defined as a change of between −3% and +3% from the previous measurement, assessed at the 0-1-year and 1-3-year intervals.

BTMs

Serum CTX and P1NP were measured using consistent immunoassay methodology throughout the study period. The P1NP:CTX ratio was calculated as an additional composite marker reflecting the balance between bone formation and resorption. Pre-analytical variables such as fasting status and time of collection were not standardised across all patients, which may contribute to inter-individual variability, as CTX in particular is known to exhibit significant diurnal variation and is influenced by food intake.

Statistical analysis

Data analysis was performed using Microsoft Excel, Office 365 (Microsoft Corporation, Redmond, WA, USA). Baseline characteristics were reported as mean and standard deviation (SD) for continuous variables and as frequencies and proportions (n (%)) for categorical variables. The normality of data distributions was assessed prior to the selection of correlation methods.

Correlations between variables were assessed using Pearson's correlation coefficient for normally distributed data and Spearman's rank correlation coefficient for skewed distributions. The significance of differences between groups was assessed using the independent samples t-test, with a threshold of p < 0.05 for statistical significance. The strength of correlation coefficients was interpreted using this classification: ±1 was considered "perfect," ±0.8-0.9 "very strong," ±0.6-0.7 "moderate," ±0.3-0.5 "fair," ±0.1-0.2 "poor," and 0 "none". The dependent (paired) samples t-test was used to compare mean changes in variables within groups over time.

Outcome 1: General BTM-BMD Relationship

The primary analysis examined correlations between changes in BTMs (CTX, P1NP, and the P1NP:CTX ratio) and changes in BMD at the hip, lumbar spine, and wrist at the one- and three-year follow-up.

Outcome 1: Grouped Analysis

A grouped analysis was performed to test for associations between changes in CTX and BMD response categories. Patients were categorised based on the magnitude of BMD change at each of the three skeletal sites (hip, spine, and wrist), creating three sets of two groups: an "Improved Group" (defined as > +0.03 g/cm² change, representing clinically significant improvement) and a "Stable Group" (defined as any change between −0.03 and +0.03 g/cm²). Baseline characteristics between groups were compared using the independent samples t-test for continuous variables and the chi-squared test for categorical variables.

Outcome 2: Medication Subgroup Analysis

The same analytical framework was applied to a subgroup analysis comparing patients treated with oral bisphosphonates and those treated with ZA. Correlations between BTM changes and BMD changes were assessed within each medication group.

## Results

This study included 214 patients with a clinical or radiological diagnosis of osteoporosis, with baseline characteristics described in Table 1. The average patient age was 67.9 (±6.3) years; 192 (90%) were female, and 166 (90%) of them were post-menopausal. Many patients had underlying secondary causes of osteoporosis, including 42 (20%) patients with contributing medical conditions (most commonly hyperparathyroidism and diabetes mellitus), 71 (33%) patients taking osteoporosis-inducing medications, 87 (41%) patients carrying a smoking history of >10 years and 6 (4.4%) patients having a BMI <18.5 kg/m². Regarding treatment regimens, 134 (63%) patients initially received ZA, and 61 (29%) patients received oral bisphosphonates. The remaining patients received either denosumab or teriparatide treatment but were not further analysed due to the limited sample sizes.

**Table 1:** Participant demographics and baseline characteristics * Females only ** Active is defined as approximately 30 minutes or greater ZA: zoledronic acid; HRT: hormone replacement therapy

Characteristic	Level or statistic	Sample (N=214)	N missing for variable
Age, years	Mean (SD)	67.9 (6.3)	0
Sex	Female	192 (90%)	0
	Male	22 (9.8%)	-
Years smoking	>10 years	87 (41%)	0
	<10 years	127 (59%)	-
Menopause status*	Pre-	2 (1.1%)	8
	Premature	16 (8.6%)	-
	Post-	166 (90%)	-
Activity level	Sedentary	24 (12%)	18
	Active**	172 (88%)	-
Body mass index, kg/m^2^	<18.5	6 (4.4%)	78
	18.5 to <25	40 (29%)	-
	25 to <30	42 (31%)	-
	30+	49 (36%)	-
FHx of osteoporosis	Positive	51 (24%)	0
	Negative	164 (76%)	-
Comorbid medical conditions	Hyperthyroidism	4 (1.9%)	0
	Hyperparathyroidism	8 (3.7%)	-
	Chronic liver disease	4 (1.9%)	-
	Chronic kidney disease	5 (2.3%)	-
	Immunosuppression	4 (1.9%)	-
	Inflammatory bowel disease	3 (1.4%)	-
	Diabetes	8 (3.7%)	-
	Anorexia	2 (0.9%)	-
	Rheumatoid arthritis	3 (1.4%)	-
	Malnutrition	1 (0.5%)	-
Osteoporosis-inducing drugs	Positive	71 (33%)	0
Vitamin D supplementation	Yes	181 (85%)	0
Vitamin D baseline measurement	Mean (SD)	70.9 (27.67)	8
Calcium supplementation	Yes	114 (53%)	0
Initial medication initiated	ZA	134 (63%)	0
	Oral bisphosphonates	61 (29%)	-
	Denosumab	18 (8.4%)	-
	Teriparatide	1 (0.5%)	-
Treatment changed following adverse reaction	ZA	15 (58%)	188
	Oral bisphosphonates	3 (12%)	-
	Denosumab	5 (19%)	-
	Teriparatide	2 (7.7%)	-
	Ceased all	1 (3.8%)	-
Antiresorptive treatment prior to study	HRT	18 (62%)	186
	ZA	2 (6.9%)	-
	Oral bisphosphonates	2 (6.9%)	-
	Denosumab	6 (21%)	-
	Raloxifene	1 (3.4%)	-
Average length of treatment prior to study, years	<1	1	194
	1	2	-
	2	5	-
	3	0	-
	4	2	-
	5	3	-
	>5	7	-
Treatment changed for other reason	Yes	4 (1.9%)	0

Outcome 1: initial analysis

Consistently, over 120 patients had results measured both at baseline and at one year for all BTM and BMD measures. As shown in Table 2, only spine BMD showed improvement at the one-year follow-up, while hip and wrist remained stable. Mean percentage changes in hip, lumbar spine, and wrist BMD were +2% (SD = 4.5%, n = 141), +3% (SD = 7.0%, n = 145), and +1% (SD = 7.0%, n = 117), respectively. At the three-year follow-up, hip and wrist BMD were again stable, changing by +2% (SD = 7.3%, n = 22) and −2% (SD = 4.7%, n = 18), respectively. Spinal BMD improved, increasing by +4% (SD = 8.5%, n = 21). BTMs showed a marked decrease at these same time periods. At one year, P1NP and CTX showed a mean change of −37% (SD = 55.1%, n = 116) and −42% (SD = 99.1%, n = 113), respectively. P1NP continued to decrease at the three-year mark, with a mean change of −2% (SD = 48.8%, n = 20), whereas CTX increased, with a mean change of +19% (SD = 67.7%, n = 16).

**Table 2:** Average changes in BTMs and BMD BTMs: bone turnover markers; BMD: bone mineral density; SD: standard deviation; P1NP: procollagen type I N-terminal propeptide; CTX: C-terminal telopeptide of type I collagen

Variables	Hip BMD		Lumbar BMD		Wrist BMD		P1NP			CTX			P1NP:CTX ratio		
Time period (years)	B–1	1–3	B–1	1–3	B–1	1–3	B–1	1–2	1–3	B–1	1–2	1–3	B–1	1–2	1–3
Average % Change	2%	2%	3%	4%	1%	-2%	-37%	13%	-2%	-42%	10%	19%	-11%	23%	9.1%
SD	0.05	0.07	0.07	0.08	0.07	0.05	0.55	0.49	0.51	0.99	0.49	0.68	0.43	0.52	0.37
n	141	22	145	21	117	18	116	19	20	113	18	16	111	49	16

Table 3 displays the statistical significance of these changes. Change in both hip and spine BMD at one year and of spine BMD from one to three years was statistically significant (P<0.001). The change in wrist BMD at one year (P=0.204) and the change in both hip and wrist BMD between one and three years (P=0.144 and P=0.084, respectively) were not statistically significant. P1NP and CTX changes were both significant at one year (P<0.001), but not significant from one to three years (P=0.214 and P=0.500, respectively). 

**Table 3:** Average BTMs and BMD at one and three years and significance of changes *P-value from paired t-test **Average % change calculated from individual changes of patients with results at both years, rather than the overall average change BTMs: bone turnover markers; BMD: bone mineral density; SD: standard deviation; P1NP: procollagen type I N-terminal propeptide; CTX: C-terminal telopeptide of type I collagen

Variables	Baseline		One year		Three years		One year vs. baseline			Three years vs. one year		
Outcome	N	Mean (SD)	N	Mean (SD)	N	Mean (SD)	N	**Average % change	*P-value	N	**Average % change	*P-value
P1NP	171	76.51 (±45.48)	135	38.27 (±37.50)	30	34.20 (±15.56)	116	-37.28%	<0.001	20	-2.42%	0.214
CTX	164	0.54 (±0.28)	130	0.21 (±0.14)	29	0.22 (±0.11)	113	-41.69%	<0.001	16	18.93%	0.500
Hip BMD	210	0.83 (±0.12)	141	0.84 (±0.11)	28	0.83 (±0.13)	141	1.58%	<0.001	22	1.91%	0.144
Lumbar BMD	212	1.03 (±0.17)	145	1.07 (±0.18)	28	1.11 (±0.18)	145	3.49%	<0.001	21	3.35%	0.001
Wrist BMD	177	0.75 (±0.12)	121	0.75 (±0.12)	25	0.74 (±0.14)	117	0.96%	0.204	18	-2.20%	0.084
P1NP:CTX	163	0.728 (0.281)	128	0.613 (0.225)	29	0.673 (0.201)	111	-11.2%	<0.001	16	9.1%	0.37

Correlation between change in BTMs and BMD 

**Figure 1:**
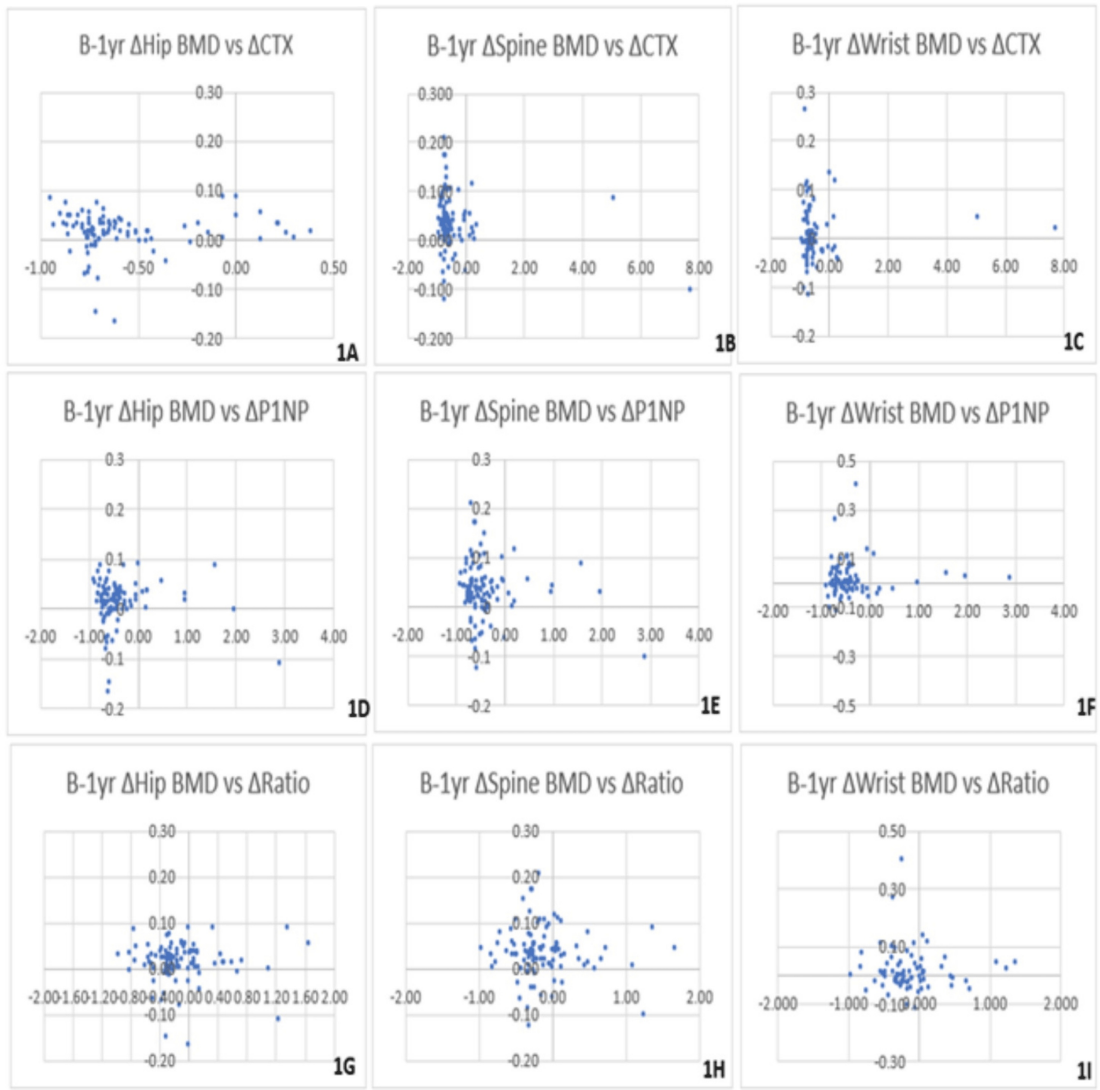
Baseline to one-year changes in BMD vs. BTMs BTMs: bone turnover markers; BMD: bone mineral density; P1NP: procollagen type I N-terminal propeptide; CTX: C-terminal telopeptide of type I collagen

These average changes in turnover markers were plotted against an average change in BMD over the same time period. The correlation coefficients (R) are displayed in Figure 1J and range from -0.143 to 0.024. The t-test for correlation showed that these values were not significant, with p-values ranging from 0.163 to 0.962. As shown in Figures 1A-C, we found that CTX consistently had weak, negative correlations with BMD at all anatomical sites (R=-0.065, -0.143, -0.012 at the hip, spine, and wrist, respectively). Thus, as BMD improved over time, CTX generally seemed to decrease, and a greater increase in BMD was weakly associated with a greater decrease in CTX. P1NP had a weak, positive correlation with BMD at the hip (R=0.005) and a weak negative correlation at the spine (R=-0.097) and wrist (R=-0.012), as shown in Figures 1D-F. It must be noted that there were quite a few patients whose results deviated significantly from the majority. Only baseline to one-year changes were compared, due to the low patient sample size.

Average change in the P1NP:CTX ratio

The P1NP and CTX ratio findings are displayed in Tables 2, 3. At the one-year follow-up, the P1NP:CTX ratio decreased, changing by -0.112 (±0.430), with 111 patients assessed. However, at the two-year mark, the ratio increased, with 49 patients showing a mean 0.233 (±0.523) change from one year and 0.019 (±0.333) at three years compared to two years. Overall, the mean change from one year to three years was 0.091 (±0.371), based on the results from 16 patients.

Correlation between the change in ratio and the change in BMD

Figures 1G-I display the weak negative correlations found between the change in the P1NP:CTX ratio and BMD at the spine (-0.04) and wrist (-0.038) and the weak positive correlation at the hip (R=0.024). Again, these correlations were not significant (P=0.703, 0.744, and 0.823, respectively).

Table 4 presents the correlation coefficients between changes in BTMs (CTX, P1NP, and the P1NP/CTX ratio) and changes in BMD at the hip, spine, and wrist from baseline to one year. All correlation coefficients are very weak (r-values ranging from −0.143 to 0.024). The correlation coefficients at the spine (r = 0.04) and wrist (r = 0.038) become slightly positive, suggesting a very faint trend where a higher formation-to-resorption balance is associated with marginal BMD gains at these sites. The hip shows a negligible negative correlation (r = −0.024).

**Table 4:** Correlation coefficients from baseline to one year P1NP: procollagen type I N-terminal propeptide; CTX: C-terminal telopeptide of type I collagen

Variable	CTX	P1NP	P1NP/CTX ratio
Hip	-0.065	0.005	-0.024
Spine	-0.143	-0.097	0.04
Wrist	-0.012	-0.012	0.038

The entire patient cohort was divided into two subgroups based on the change in BMD, as shown in Table 5. Group 1 was “Improved” (patients with ≥3% increase in BMD) and group 2 was “Stable” (patients with -3% to <+3% change in BMD). The two groups were comparable in all aspects of their baseline characteristics (p>0.05) for groups divided based on hip, spine, and wrist BMD. Baseline BMD at all sites, P1NP, CTX, and the P1NP:CTX ratio were also similar between the two groups (p>0.05).

**Table 5:** Stable vs. improved ∆BMD (hip) subgroup analysis BMI: body mass index; BMD: bone mineral density; SD: standard deviation; P1NP: procollagen type I N-terminal propeptide; CTX: C-terminal telopeptide of type I collagen

Variable	Improved group count (n=50)	Improved group mean/proportions	Stable group count (n=76)	Stable group mean/proportions	P-value
Age (y)	50	69.08 (±5.79)	76	68.03 (± 4.38)	0.11
BMI	33				0.14
BMI (kg/m^2^) <18.5	3	0.06	0	0.00	
BMI (kg/m^2^) 18.5–<25	11	0.22	14	0.18	
BMI (kg/m^2^) 25–<30	8	0.16	16	0.21	
BMI (kg/m^2^) >30	11	0.22	21	0.28	
Menopausal status	46	-	64	0.84	0.42
Post-menopausal	41	0.82	56	0.74	
Premature menopause	1	0.02	0	0.00	
Premenopausal	4	0.08	8	0.11	
Smoking status	48	-	73	0.96	0.19
>10 smoking years	16	0.32	32	0.42	
<10 smoking years	32	0.64	41	0.54	
Family history of osteoporosis	10	0.20	20	0.26	0.44
Osteoporosis-inducing drugs	9	0.18	24	0.32	0.95
Hyperthyroidism	2	0.04	0	0.00	-
Hyperparathyroidism	2	0.04	3	0.04	-
Chronic liver disease	0	0.00	1	0.01	-
Chronic kidney disease	2	0.04	3	0.04	-
Immunosuppression	1	0.02	2	0.03	-
Inflammatory bowel disease	0	0.00	2	0.03	-
Diabetes	1	0.02	1	0.01	-
Anorexia	2	0.04	0	0.00	-
Rheumatoid arthritis	0	0.00	3	0.04	-
Malnutrition	0	0.00	0	0.00	-
Baseline 25(OH)D (ng/mL)	-	73.98 (±29.99)	-	69.32 (±24.12)	0.35
25(OH)D Supplementation	42	0.840	59	0.78	0.38
P1NP (ng/mL)	-	81.85 (±67.73)	-	74.14 (±35.57)	0.45
∆P1NP (ng/mL)	-	-0.38 (±0.53)	-	-0.43 (±0.45)	0.65
CTX (ng/mL)	-	0.59 (±0.36)	-	0.53 (±0.24)	0.44
∆CTX (ng/mL)	-	-0.43 (±1.01)	-	-0.55 (±0.30)	0.41
Calcium supplementation	27	0.54	26	0.34	-

Significance of difference in BTMs/BMD/ratio changes between the BMD improved and stable groups 

As shown in Table 6, the t-test for comparison of means was used to compare ∆CTX from one to three years for those with improvement in BMD (Improved Group) and those with stable BMD (Stable Group). The ∆CTX in Group 1 compared to Group 2 was -0.43 (±1.01) compared to -0.55 (±0.24); p=0.41. Similarly, ∆P1NP was -0.38 (± 0.53) in Group 1 compared to -0.43 (±0.45) in Group 2; p=0.65. Significant values were consistently p>0.05 in each subgroup analysis for both the spine and radius. Correlation between groups with regard to change in BTMs and change in BMD was also assessed for the ‘baseline to one year’ time periods. Correlation coefficients and p-values for ∆CTX were R=0.01 (p=0.95), R=0.19 (p=0.34), and R=0.44 (p=0.09) at the hip, spine, and wrist, respectively. Thus, correlations were persistently weak and not significant, indicating that clinically significant improvements in BMD did not consistently map to decreased BTMs. Findings for P1NP were similar, as shown in Table 6.

**Table 6:** Correlation coefficient and correlation significance for change in BTMs in subgroup analysis BTMs: bone turnover markers; P1NP: procollagen type I N-terminal propeptide; CTX: C-terminal telopeptide of type I collagen

Variables	∆P1NP	∆P1NP(P)	∆CTX	∆CTX(P)
Hip	-0.23	0.25	0.01	0.95
Spine	0.02	0.92	0.19	0.34
Wrist	0.19	0.49	0.44	0.09

Outcome 2: ZA and oral bisphosphonates analysis

Table 7 shows the one-year follow-up in ZA patients with an average of 44% suppression in CTX (n=78) and 39% suppression in P1NP (n=79). There was a 1.7% increase in hip (n=93) and wrist BMD (n=82) and a 4% increase in spine BMD (n=95). All of which were statistically significant (p<0.05) using a paired t-test, except the wrist (n=82) BMD scores.

**Table 7:** Change in BTMs and BMD across all three sites over one year split across ZA and oral bisphosphonate groups BTMs: bone turnover markers; BMD: bone mineral density; P1NP: procollagen type I N-terminal propeptide; CTX: C-terminal telopeptide of type I collagen; ZA: zoledronic acid

Zoledronic acid baseline to one year		Oral bisphosphonates baseline to one year	
∆CTX (p)	44% suppression (<0.001)	∆CTX (p)	57% suppression (<0.001)
∆P1NP (p)	39% suppression (<0.001)	∆P1NP (p)	44% suppression (<0.001)
∆BMD hip (p)	1.7% (<0.001)	∆BMD hip (p)	0.9% (0.47)
∆BMD spine (p)	4% (<0.001)	∆BMD spine (p)	3% (0.06)
∆BMD wrist (p)	1.7% (0.06)	∆BMD wrist (p)	-1.5% (0.11)

Table 7 further shows the one-year follow-up in oral bisphosphonate patients with an average of 57% suppression in CTX (n=29) and 49% suppression in P1NP (n=30). At the hip (n=36), there was a 0.9% increase in BMD, a 3% increase at the spine (n=40), and a 1.5% decrease at the wrist (n=26). BTMs were statistically significant (p<0.05), while all BMD results were insignificant.

Table 8 shows an analysis of variance of 83 patients with follow-up CTX values, which showed an average of 59% suppression and a variance of 894%. Table 8 further shows 87 patients with follow-up P1NP values, which showed an average of 41% suppression and a variance of 3168%.

**Table 8:** Analysis of variance of BTMs across all patients with one-year follow-up BTMs: bone turnover markers; P1NP: procollagen type I N-terminal propeptide; CTX: C-terminal telopeptide of type I collagen

Groups	Count	Sum	Average	Variance
CTX	83	4898.60735	59.0193657	894
P1NP	87	3584.62172	41.2025485	3168

**Figure 2:**
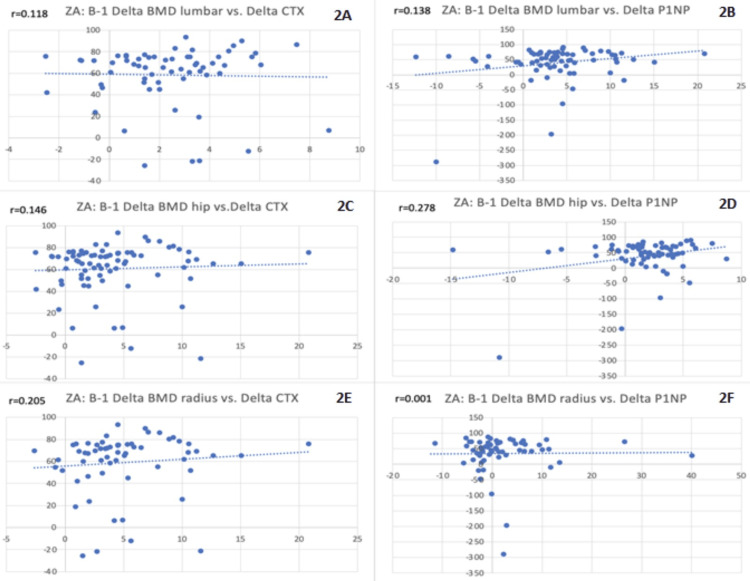
Baseline to one year changes in BMD vs. BTMs for ZA-treated patients BTMs: bone turnover markers; BMD: bone mineral density; P1NP: procollagen type I N-terminal propeptide; CTX: C-terminal telopeptide of type I collagen; ZA: zoledronic acid

Figure 2 shows ZA-treated patients and their BMD values at three sites plotted against both BTMs. At the one-year follow-up, CTX and P1NP were both found to be suppressed with rising BMD levels, with poor, positive correlations everywhere except the wrist in the oral bisphosphonate group, wherein the majority of patients had less than 0% BMD change; this is further addressed in the discussion. All these correlations were tested using the t-test for correlation and were found to be insignificant. The figure below displays the general trend that these scatterplots followed, with most patients being in the right upper quadrant.

Table 9 shows baseline characteristics for patients treated with ZA split into positive and stable BMD change. Categorical variables, baseline BMD at all sites, and P1NP and CTX values were also similar between the two groups (p>0.05), making further analyses between the groups valid, as the groups were able to be compared.

**Table 9:** ZA subgroup demographics and baseline results. Subgroups have been split into patients, as done in Table 5 P-values were calculated using an independent samples t-test with similar variance (continuous) and chi-square (categorical). Categories with 0 patients did not have calculated p-values BMD: bone mineral density; SD: standard deviation; P1NP: procollagen type I N-terminal propeptide; CTX: C-terminal telopeptide of type I collagen; ZA: zoledronic acid; BMI: body mass index

Variable	Positive BMD ZA count	Positive BMD ZA mean/proportions	Stable BMD ZA count	Stable BMD ZA mean/proportions	P-value
Age (y)	35	68.9 (±6.1)	51	67.2 (±6.6)	-
BMI	22	0.113	35	0.179	0.91
BMI (kg/m²) <18.5	1	0.005	0	0.000	
BMI (kg/m²) 18.5–<25	7	0.036	9	0.046	
BMI (kg/m²) 25–<30	6	0.031	12	0.062	
BMI (kg/m²) >30	8	0.041	14	0.072	
Menopausal Status	33	0.169	44	0.226	0.43
Post-menopausal	30	0.154	37	0.190	
Premature Menopause	2	0.010	7	0.036	
Premenopausal	1	0.005	0		
Smoking Status (Ex or current)	13	0.067	26	0.133	0.6
>10 Smoking Years	9	0.046	20	0.103	
<10 Smoking Years	4	0.021	6	0.031	
Family History of Osteoporosis	8	0.041	15	0.077	0.5
Osteoporosis-Inducing Drugs	11	0.056	20	0.103	0.46
Hyperthyroidism	1	0.005	0	0.000	-
Hyperparathyroidism	2	0.010	2	0.010	-
Chronic Liver Disease	0	0.000	1	0.005	-
Chronic Kidney Disease	1	0.005	3	0.015	-
Immunosuppression	1	0.005	1	0.005	-
Inflammatory Bowel Disease	0	0.000	0	0.000	-
Diabetes	1	0.005	1	0.005	-
Anorexia	1	0.005	0	0.000	-
Rheumatoid Arthritis	0	0.000	3	0.015	-
Malnutrition	0	0.000	0	0.000	-
Spine BMD (g/cm²)	35	1.06 (±0.17)	51	1.04 (±0.15)	0.306
Hip BMD (g/cm²)	35	0.828 (±0.083)	51	0.85 (±0.12)	0.184
33% Radius BMD (g/cm²)	30	0.73 (±0.105)	46	0.76 (±0.14)	0.120
Baseline 25(OH)D (ng/mL)	35	76.29 (±32.109)	50	67.82 (±22.37)	0.092
25(OH)D Supplementation	31	0.16	39	0.2	0.16
P1NP (ng/mL)	30	85.83 (±74.35)	43	82.77 (±36.90)	0.418
∆P1NP (% of suppression)	28	42.37% (±44.71)	37	43.3% (±45.71)	0.467
CTX (ng/mL)	29	0.57 (±0.373)	43	0.57 (±0.24)	0.491
∆CTX (% of suppression)	26	58.27% (±33.99)	37	58.69% (±22.09)	0.478
Calcium supplementation	19	0.097	19	0.097	0.12
∆P1NP:CTX ratio	-	0.73	-	0.74	-

Table 10 shows baseline characteristics for patients taking oral bisphosphonates split into positive and stable BMD change, defined above. Categorical variables were similar between the two groups (P>0.05); however, baseline results for various BMD and BTM results were significantly different (P<0.05). This will be further explained in the discussion. After further subgroup division, the bisphosphonate subgroups had less than 30 patients in many categories, making the analysis imprecise; consequently, it is difficult to draw valid conclusions.

**Table 10:** Oral bisphosphonate subgroup demographics and baseline results. Subgroups split into patients as in Table 5 P-values were calculated using an independent samples t-test with similar variance (continuous) and chi-square (categorical). Categories with 0 patients did not have calculated P-values BMD: bone mineral density; SD: standard deviation; P1NP: procollagen type I N-terminal propeptide; CTX: C-terminal telopeptide of type I collagen; ZA: zoledronic acid; BMI: body mass index

Variable	Positive BMD BP count	Positive BMD BP mean/proportions	Stable BMD BP count	Stable BMD BP mean/proportions	P-value
Age (y)	11	67.36 (±3.7)	19	67.63 (±6.3)	-
BMI	8	0.041	12	0.062	0.83
BMI (kg/m²) <18.5	2	0.010	0	0.000	
BMI (kg/m²) 18.5-<25	3	0.015	3	0.015	
BMI (kg/m²) 25-<30	2	0.010	3	0.015	
BMI (kg/m²) >30	1	0.005	6	0.031	
Menopausal Status	10	0.051	18	0.092	0.24
Post-menopausal	8	0.041	17	0.087	
Premature Menopause	2	0.010	1	0.005	
Premenopausal	0	0.000	0	0.000	-
Smoking Status (Ex or current)	6	0.031	10	0.051	0.7
>10 Smoking Years	5	0.026	9	0.046	
<10 Smoking Years	1	0.005	1	0.005	
Family History of Osteoporosis	2	0.010	3	0.015	0.87
Osteoporosis-Inducing Drugs	2	0.010	2	0.010	0.55
Hyperthyroidism	0	0.000	0	0.000	-
Hyperparathyroidism	0	0.000	1	0.005	-
Chronic Liver Disease	0	0.000	0	0.000	-
Chronic Kidney Disease	0	0.000	0	0.000	-
Immunosuppression	0	0.000	0	0.000	-
Inflammatory Bowel Disease	0	0.000	1	0.005	-
Diabetes	0	0.000	0	0.000	-
Anorexia	1	0.005	0	0.000	-
Rheumatoid Arthritis	0	0.000	0	0.000	-
Malnutrition	0	0.000	0	0.000	-
Spine BMD (g/cm²)	11	0.928 (±0.152)	19	1.06 (±0.16)	0.0202
Hip BMD (g/cm²)	10	0.743 (±0.133)	19	0.85 (±0.1)	0.01949
33% Radius BMD (g/cm²)	8	0.679 (±0.105)	15	0.78 (±0.1)	0.01946
Baseline 25(OH)D (ng/mL)	10	67.6 (±25.36)	19	75.37 (±29.31)	0.2331
25(OH)D Supplementation	7	0.036	15	0.077	0.36
P1NP (ng/mL)	8	84.38 (±39.32)	16	61.56 (±23.32)	0.0813
∆P1NP (% of suppression)	6	65.97% (±20.97)	11	40.34% (±51.24)	0.0841
CTX (ng/mL)	8	0.68 (±0.29)	16	0.47 (±0.22)	0.0482
∆ CTX (% of suppression)	6	81.67% (±11.28)	11	49.78% (±46.13)	0.0251
Calcium supplementation	5	0.026	4	0.021	0.16
∆P1NP:CTX ratio	-	0.81	-	0.81	-

## Discussion

Our study confirmed the concurrent increases in BMD and decreases in BTMs known to occur during antiresorptive treatment in the short term, with significant values observed at one-year follow-up. BMD and BTM stability, rather than statistically significant improvement, were noted at three years compared to one-year values. Effects of therapy were most notable at the lumbar spine, followed by hip and then wrist BMD, with the latter showing no significant change at either follow-up point.

There was no significant association between BMD and either the BTMs or the P1NP:CTX ratio in the subgroup analysis comparing oral and IV bisphosphonate therapy, with no discernible trend or significant correlation.

General changes and correlations between BTMs and BMD

In our study, BTMs and all BMD measures showed statistically significant short-term improvements following treatment. When measured at the one-year mark, P1NP and CTX both decreased, and BMD increased. These findings are consistent with those of multiple studies [32-35], which have shown an increase in BMD occurring concurrently with decreased BTMs.

BTM changes from one to three years were not significant. Although difficult to interpret due to the limited sample size, multiple studies, including the TRIO study, have also noted that the most significant changes in BTMs occur early in treatment [36]. Additional studies have also demonstrated the correlation between a statistically significant rise in BTMs and an increase in fracture risk [37-39]. Thus, long-term maintenance of initially reduced BTMs, without a statistically significant increase or decrease, as was found in our study, could still indicate an effective therapeutic response.

In our study, the only statistically significant BMD increase from one to three years occurred at the lumbar spine. Hip and wrist BMD both remained clinically stable, a positive outcome, as stability is one of the main goals in treatment [40]. The limited number of patients with documented wrist BMD at three years complicates the interpretation of these findings. However, overall, it appears that a sustained overall decrease in BTMs may be linked to continued BMD stability or improvement at both one and three years, in the order of spine, hip, and then wrist.

Correlation analysis

In our study, although BTMs and the ratio decreased (indicating greater formation than resorption) at both one and three years, the correlation with ∆BMD was weak and not statistically significant. In contrast, Trento et al. [41] reported a statistically significant inverse correlation between serum CTX and BMD at the spine and femur. A potential confounder in both studies is the significant inter-individual variability in CTX levels. Many patients had outlier results (Figure 1), potentially due to poor medication compliance, suboptimal absorption, or undetected cases of secondary osteoporosis [42]. An additional contributor to inter-individual BTM variability may be prior anti-resorptive therapy. Patients previously treated with denosumab may exhibit rebound elevation of BTMs, particularly CTX, following cessation, while those with prior bisphosphonate exposure may demonstrate atypically suppressed baseline BTMs due to the prolonged residual effect of these agents on the skeleton. In the present cohort, the small number of patients with prior anti-resorptive use (e.g., n = 5 for prior denosumab) precluded formal subgroup analysis; however, this represents an important confounder that future prospective studies should account for.

Previous studies found significant correlations between BTMs and BMD at early follow-up. For example, Schini et al. [33] found significant correlations between markers and BMD at one and six months post-treatment initiation in patients with glucocorticoid-induced osteoporosis. Thus, a shorter follow-up period may have yielded stronger correlation findings in our study, highlighting a future need to investigate the use of BTMs as early indicators of therapeutic efficacy.

In our study, the P1NP:CTX ratio initially decreased at the one-year mark and then increased at two and three years compared to one year. The initial decrease contradicts multiple studies [40,43,44] wherein an increased ratio is logical, given that bone resorption must be inhibited more than bone formation to increase BMD. The contradictory one-year ratio decrease in our study may be due to the inclusion of mostly patients with a recent MTF in our study, as the ratio is known to decrease post-fracture [33,45], and markers of resorption increase much more quickly than those of formation [46].

In our study, the remaining trend in the P1NP:CTX ratio was that of a mild and insignificant increase at two and three years; the correlation with BMD improvement was weak and not significant. Thus, both measures of BTM change and the P1NP:CTX ratio appear to be of limited value in assessing BMD improvement during antiresorptive therapy, especially when treatment is initiated post-fracture.

Improved vs. stable BMD change subgroup analysis

We performed a subgroup analysis comparing patients with clinically significant increases in BMD and those with BMD stability. Our study showed no significant link between baseline characteristics, such as menopausal status or BMI, and patient response to treatment at one year (as reflected in BTM or BMD response). In line with findings by Helynen et al. [44], we found that mean initial BTMs did not differ between groups. However, other studies have found that high pretreatment levels of CTX and P1NP predicted greater BMD improvement at the hip [44,47]. Thus, there remains a significant discrepancy in findings.

Importantly, there was no significant difference between groups regarding change in either P1NP or CTX. Subgroup analysis based on spine and wrist BMD yielded similar findings. It may be beneficial to compare patients with BMD improvement to those with decline, as BMD decrease is a characteristic of failed treatment and an indication for switching therapies [40].

Outcome 2: BMD, BTMs, and correlation across ZA and oral bisphosphonate subgroups

In our study, BTMs were suppressed at the hip and spine at the one-year follow-up across both medication groups. Both BTMs had a significant degree of suppression in comparison to other studies. A consensus statement by Wu et al. reported a threshold of efficacy in bisphosphonate treatment as at least a 30% decrease from baseline for CTX and at least 20% for P1NP [48]. Across our ZA group, we observed 44% and 39% suppression in CTX and P1NP, respectively, at one year, thus reaching the required least significant change (LSC). All sites showed an increase in BMD with BTM suppression. However, these correlations were poor and not statistically significant. This is in keeping with studies which report an association but an insignificant correlation between hip BMD and P1NP, and no independent relationship between BTMs and fracture risk [38,48]. Thus, the role of BTMs appears limited to acting as an adjunct to a BMD assessment of bone health.

At one year, we found a decreased P1NP:CTX ratio amongst ZA patients, indicating greater suppression in CTX than P1NP. This reflects the expected decrease in osteoclastic activity and is in line with findings by Wei et al. [34] and Gossiel et al. [49]. Thus, in medicated patients, adequate suppression of CTX may be more useful than P1NP in monitoring BMD response. However, interpretation is limited due to BTM variability and imprecise assays, and BTMs and the P1NP:CTX ratio require further exploration [50]. We also found that subgroup analysis comparing ZA-treated patients with positive BMD change and stability showed no correlation between the degree of change in BTMs and BMD. Thus, amongst ZA-treated patients, it may be more relevant to assess attainment of LSC rather than the degree of BTM suppression in predicting BMD response [51].

In patients taking oral bisphosphonates, we observed 57% and 44% suppression of CTX and P1NP, respectively. As for ZA-treated patients, this was significant compared to the LSC. However, correlations with BMD were weak and not significant. BMD at the hip and spine increased but decreased at the wrist. Once again, this is consistent with the discordance in wrist BMD measurements. Additionally, unlike ZA-treated patients, the oral bisphosphonate group had a poor BMD response to medication and insignificant change at all sites. This can be attributed to short follow-ups and low sample size. There was also no assessment of adherence to oral bisphosphonates. There is also the possibility that inadequate vitamin D optimisation during treatment is a potential contributor to variable BMD response and represents a factor that warrants further investigation, particularly at peripheral sites such as the wrist. However, our cohort at one and three years saw approximately eight patients sustain a decrease in vitamin D, with 85% (n=181) of patients receiving supplementation.

In the subgroup analysis comparing positive change to stable BMD, patients with positive BMD change had a much greater magnitude of BTM change, specifically, a change in CTX. This is in keeping with the findings of Bauer et al., who concluded that greater change in one or more BTMs was associated with fewer fractures and inferred an increase in BMD [26]. Thus, long-term maintenance of initially reduced BTMs, without a statistically significant increase or decrease, as was found in our study, could still indicate an effective therapeutic response. This was also inferred by the MOBILE trial, which found a significant association between serum CTX suppression measured at three months and BMD one-year response at the lumbar spine [52]. Once again, as BTM values were taken at the same time as BMD, our study was unable to assess the short-term predictive value of BTMs. However, since longer-term values remained suppressed, there may be an association between a positive BMD change and the magnitude of serum CTX suppression. Compared to ZA-treated patients, the ratio of P1NP:CTX suppression was also decreased but to a lesser degree, indicating greater suppression of CTX or lesser suppression of P1NP, which reflects stability or an increase in bone mass. These findings are quite varied and indicate areas for future investigation of the P1NP:CTX ratio.

Strengths and limitations

This study assessed the clinical role of BTMs and the P1NP:CTX ratio, comparing them to the gold standard measurement of bone health, BMD. A recent consensus report concluded that BTMs would be useful for monitoring and predicting response to bisphosphonate therapy [52]. However, our study found that trends in BTMs differ for different anatomical locations and are difficult to interpret in longer-term therapy. Oral and IV bisphosphonate therapy effects on both BMD and BTMs were also compared head-to-head. The retrospective study design allowed assessment of a broader, more realistic treatment population.

The retrospective design meant, however, an inability to establish causation. A significant limitation of this study was loss to follow-up, which substantially reduced available sample sizes at the three-year time point. This attrition was attributable to non-adherence, missed appointments, patient relocation, medication changes, discharge to primary care, and death, and represents a major constraint on the conclusions that can be drawn from three-year data. Inconsistent and infrequent data collection timepoints further limited adequate sample sizes and impeded the analysis of temporal relationships, particularly over short-term follow-up. Long-term therapy could not be assessed. Limited patients treated with denosumab and teriparatide prevented the investigation of these other treatments. Data inconsistency was contributed to by treatment changes during the study (n=23) and variations in the pathology centre and anatomical location of hip and lumbar spine BMD measurements. Fan et al. have shown homogeneity in hip BMD measurements at the femoral neck and total hip, but there is a discrepancy in varying sites of spine BMD measurement [30,53]. This, and the inclusion of patients already on treatment, potentially contributed to the outliers and could have skewed the data. To moderate the effects of outliers, Spearman’s correlation was utilised for skewed data distributions [54]. Different centres use different scanners, potentially causing inconsistency in values. However, most of our patients used the same scanner (Lunar System, n=208 (97.2%); Hologic System, n=6 (2.8%)), thus ameliorating this effect.

A further limitation of this study is that the influence of prior anti-resorptive therapy on baseline and follow-up BTM levels could not be fully assessed. Although prior anti-resorptive use was recorded, the number of patients with prior exposure was insufficient for subgroup correlation analysis; for instance, only five patients had prior denosumab use. Prior denosumab discontinuation is associated with a well-described rebound rise in BTMs, while prior bisphosphonate use may result in prolonged BTM suppression independent of current therapy. Both effects represent potential confounders in the interpretation of BTM trajectories observed in this cohort, and this warrants investigation in future studies with larger and more treatment-diverse populations.

## Conclusions

Our study found that BTMs were suppressed with therapy concurrently with the expected increase in BMD at one year, and both markers mostly showed stability at three years. There was poor correlation between BTMs and BMD, and minimal differences in baseline and degree of change in BTMs between patients with increased BMD and those with stable BMD. The P1NP:CTX ratio was suppressed at two and three years post-fracture, but it was not statistically significant, and it showed no significant correlation with BMD change. These markers did, however, show potential for accurately reflecting intervention outcomes early and indicating the likelihood of treatment efficacy. These findings were consistent in our subgroup analysis of oral bisphosphonate and ZA treatment. Overall, there remain challenges to the interpretation of BTMs in osteoporosis, and further research into their role in reflecting treatment efficacy is required.
